# More Cyberbullying, Less Happiness, and More Injustice—Psychological Changes During the Pericyberbullying Period: Quantitative Study Based on Social Media Data

**DOI:** 10.2196/64451

**Published:** 2025-02-25

**Authors:** Xingyun Liu, Miao Liu, Xin Kang, Nuo Han, Yuehan Liao, Zhihong Ren

**Affiliations:** 1 Key Laboratory of Adolescent Cyberpsychology and Behavior Ministry of Education Wuhan China; 2 Key Laboratory of Human Development and Mental Health of Hubei Province School of Psychology Central China Normal University Wuhan China; 3 Department of Psychology Faculty of Arts and Sciences Beijing Normal University at Zhuhai Zhuhai China; 4 Beijing Key Laboratory of Applied Experimental Psychology Faculty of Psychology Beijing Normal University Beijing China

**Keywords:** cyberbullying, pericyberbullying period, social media, well-being, morality, suicide risk, personality traits

## Abstract

**Background:**

The phenomenon of cyberbullying is becoming increasingly severe, and many studies focus on the negative psychological impacts of cyberbullying survivors. However, current survey methods cannot provide direct and reliable evidence of the short-term psychological effects of cyberbullying survivors, as it is impractical to measure psychological changes before and after such an unpredictable event in a short period.

**Objective:**

This study aims to explore the psychological impacts of cyberbullying on survivors during the pericyberbullying period, defined as the critical time frame surrounding the first cyberbullying incident, encompassing the psychological changes before, during, and after the event.

**Methods:**

We collected samples from 60 cyberbullying survivors (experimental group, 94/120, 78% female) and 60 individuals who have not experienced cyberbullying (control group, matched by sex, location, and number of followers) on Sina Weibo, a social media platform developed by Sina Corporation. During the pericyberbullying period, we retrospectively measured psychological traits 3 months before and after the first cyberbullying incident for both groups. Social media data and predictive models were used to identify survivors’ internal psychological traits, including happiness, suicide risk, personality traits, and moral perceptions of the external environment. Network analysis was then performed to explore the interplay between cyberbullying experiences and psychological characteristics.

**Results:**

During the pericyberbullying period, survivors exhibited significantly reduced happiness (*t*_59_=2.14; *P*=.04), marginally increased suicide risk, and significant changes in the Big 5 personality traits, including decreased conscientiousness (*t*_59_=2.27; *P*=.03), agreeableness (*t*_59_=2.79; *P*=.007), and extraversion (*t*_59_=2.26; *P*=.03), alongside increased neuroticism (*t*_59_=–3.42; *P*=.001). Regarding moral perceptions of the external environment, survivors showed significant increases in communicative moral motivation (*t*_59_=–2.62; *P*=.011) and FairnessVice (*t*_59_=–2.20; *P*=.03), with a marginal rise in PurityVice (*t*_59_=–1.88; *P*=.07). In contrast, the control group exhibited no significant changes during the same time frame. Additionally, network analysis revealed that beyond cyberbullying experiences, core psychological characteristics in the network were neuroticism, conscientiousness, and Oxford Happiness.

**Conclusions:**

By leveraging noninvasive retrospective social media data, this study provides novel insights into the short-term psychological impacts of cyberbullying during the pericyberbullying period. The findings highlight the need for timely interventions focusing on enhancing survivors’ happiness, reducing suicide risk, adjusting personality traits, and rebuilding moral cognition to mitigate the negative effects of cyberbullying.

## Introduction

### Background

Cyberbullying is a growing global phenomenon, with the prevalence of cyberbullying experiences increasing significantly over the past 2 decades [1]. In today’s highly interconnected world, anyone can become a survivor of cyberbullying [2]. Cyberbullying refers to the use of digital technologies to intentionally harm others, either directly or indirectly. When the survivor perceives the perpetrator’s intent as deliberate and experiences harm, they are considered a survivor of cyberbullying [3].

Cyberbullying is a significant source of social stress [4]. According to the stress theory proposed by Lazarus and Folkman [5] and Selye’s [6] general adaptation syndrome, individuals who perceive themselves as unable to cope with stress are more likely to experience negative psychological outcomes. In the context of cyberbullying, survivors often experience intense psychological stress in the short term, which can affect their happiness, suicide risk, personality traits, and perceptions of moral injustice in the external environment [7-19].

Although previous studies have demonstrated the negative psychological impacts of cyberbullying, they have largely relied on self-report questionnaire methods, which are susceptible to individual recall bias. Moreover, a few studies have investigated the psychological characteristics of individuals both before and after experiencing cyberbullying, which may obscure the effects of innate differences as well as the role of cyberbullying itself [20]. Additionally, existing longitudinal studies typically focus on the long-term impacts that include data collected prior to cyberbullying, with research time periods ranging from 6 months to 3 years [21]. Due to the unpredictability of cyberbullying, collecting a sufficient sample to assess its immediate psychological impacts presents significant challenges. To address this issue, this study draws inspiration from analogous terms in the medical field, such as the “perinatal period” and the “perioperative period,” which describe key stages of change surrounding specific events or states [22]. Based on these concepts, the “pericyberbullying period” is defined as a critical time frame that encompasses the psychological fluctuations before the occurrence of a cyberbullying event, the immediate reactions during the event, and the adaptation process following its conclusion. The pericyberbullying period provides a new lens through which to explore the short-term and immediate psychological impacts of cyberbullying on survivors. It helps to reveal the dynamic psychological changes associated with cyberbullying with greater precision, while also offering theoretical support for the development of time-based intervention strategies. By leveraging the richness, openness, timeliness, and high traceability of social media data [23], this study enables a retrospective analysis of the psychological changes survivors experience during the pericyberbullying period. Previous studies have used social media data, such as the online ecological recognition method, to retrospectively investigate the psychological effects of negative events (eg, the loss of an only child and domestic violence) [20,24]. This study applies this method innovatively to cyberbullying research, further validating and expanding the potential of social media data in psychological studies.

#### Cyberbullying Experiences and Well-Being

Positive psychology posits that well-being, as a complex, multifaceted, and central positive experience, is a multidimensional psychological structure [25]. Subjective well-being (SWB), based on the hedonistic perspective, primarily includes classical indicators such as positive and negative emotions [26]. In contrast, psychological well-being (PWB), grounded in the eudaimonic perspective, comprises 6 dimensions: self-acceptance, life purpose, positive relationships, environmental mastery, personal growth, and autonomy [27]. Integrating both SWB and PWB, researchers have proposed the unidimensional Oxford Happiness Questionnaire [28]. High levels of well-being are closely associated with self-actualization, coping with life stress, and finding meaning in life [29]. Health is considered the foundation of happiness, as individuals can only experience higher levels of well-being when they are in good physical and mental condition [30].

Recent studies have found that cyberbullying experiences can predict various subsequent psychological outcomes, such as negative emotions, disrupted self-evaluation and value systems, negative interpersonal relationships, and loss of control, all of which can impair an individual’s well-being [31,32]. Specifically, cyberbullying experiences are often accompanied by negative emotions. Research by Zhao et al [13] found that cyberbullying experiences can trigger low thresholds, high emotional intensity, and slow recovery to baseline negative emotions in adolescents. These emotions can mediate the relationship between cyberbullying experiences and non-suicidal self-injury, which is significantly negatively correlated with well-being [17]. When an individual is bullied, their self-evaluation system and sense of self-worth are disrupted, leading to a decreased level of self-acceptance and the development of a negative self-concept [9,11]. Self-acceptance is an important dimension of PWB. Research has found that self-acceptance is a predictor of psychological distress and can protect individuals from the effects of depression [13]. Fang et al [10] found that self-acceptance mediates the relationship between cyberbullying experiences and depression in college students.

Cyberbullying experiences also affect social behavior. Survivors of cyberbullying often experience high levels of social anxiety [33,34], and low self-esteem and self-acceptance can negatively impact interpersonal relationships [10]. Audrin and Blaya [35] found that factors such as the repetitive nature of web-based behavior, the anonymity of cyber aggressors, and the large audience can damage the well-being of cyberbullying survivors. The anonymity, large audience, and lack of spatial and temporal limitations of cyberbullying increase the difficulty of controlling these incidents, reducing individuals’ psychological sense of control [36]. When traumatic events are uncontrollable, even individuals with high perceived control may experience posttraumatic stress disorder symptoms, while those with relatively low control are more likely to experience posttraumatic stress disorder symptoms [8].

#### Cyberbullying Experiences and Suicide Risk

Suicide risk is closely related to lower PWB [37]. According to the suicide stress theory [38], cyberbullying experiences increase suicide risk through several psychological mechanisms, including value conflict, the gap between ideals and reality, and relative deprivation stress [39]. Studies indicate that cyberbullying can significantly increase suicide-related behaviors, including suicidal ideation, especially in adolescents [7,12]. Longitudinal studies also show that early cyberbullying experiences can lead to later suicidal thoughts and behaviors, with core self-evaluation serving as a mediator between the two [40].

#### Cyberbullying Experiences and Personality Traits

Personality traits and life events are intertwined [24]. On one hand, personality differences exist before life events occur, leading to different probabilities of experiencing certain life events, known as the “selection effect” [41]. On the other hand, personality can change in response to life events, known as the “socialization effect” [42]. Significant life events, especially those occurring for the first time, may have a more pronounced impact, such as the death of a spouse, first-time unemployment, or first-time domestic violence, compared to similar later events [24].

Existing research primarily measures the relationship between personality and life events (eg, cyberbullying experiences) through relatively long (eg, 1 year or even 2 years), fixed-interval self-reports, and compares personality differences between groups with and without specific life events. However, the relationship between cyberbullying experiences and personality traits is unclear. It is uncertain whether certain personality traits lead to cyberbullying experiences or if cyberbullying leads to changes in personality traits. For example, a 2-year study on Dutch individuals aged 15 years and older found that compared to individuals who have not experienced cyberbullying, cyberbullying survivors had lower conscientiousness, higher openness and neuroticism, and no significant difference in agreeableness [18]. Ryff and Keyes [27] found that Turkish student survivors of cyberbullying were positively correlated with neuroticism, agreeableness, and extraversion, and negatively correlated with conscientiousness. A study on high school students found that personality traits do not seem to be predictors of cyberbullying experiences [20]. These conflicting results may be due to limitations in current methods of measuring personality change. For example, some studies may include survivors who have experienced prolonged cyberbullying during the measurement interval, with personality changes influenced by the continuous occurrence of cyberbullying rather than the event itself. Additionally, comparing cyberbullying and noncyberbullying groups to study the effects of cyberbullying makes it difficult to distinguish between the impact of cyberbullying and the inherent differences between the groups.

#### Cyberbullying Experiences and Morality

Cyberbullying is a traumatic event for survivors, who perceive unjust treatment in their social environment, thereby disrupting their belief in a just world [16]. This disruption may lead to changes in the survivors’ moral perceptions of the external environment, causing them to perceive more unfairness. Cyberbullying experiences are a predictor of cyberbullying behavior [31]. When individuals perceive unjust treatment in their environment, they may engage in moral disengagement to alleviate guilt, making them more likely to engage in unethical behavior, such as retaliatory cyberbullying [43]. Moral behavior is typically driven by moral motivation; thus, the perception of unfairness following cyberbullying experiences may influence an individual’s moral motivation and subsequently affect their future moral behavior.

### Objectives

This study leverages social media data to explore the immediate psychological changes experienced by survivors during the pericyberbullying period, encompassing the time before and after their first encounter with cyberbullying. It comprehensively examines the short-term impacts of cyberbullying on multidimensional psychological characteristics, including well-being and its subdimensions (positive emotions, negative emotions, and self-acceptance), suicide risk and its related dimensions (shame and guilt, and self-regulation), personality traits (eg, agreeableness), and moral cognition of the external environment (eg, the FairnessVice in moral foundations and the communication in moral motivation). Additionally, it uses network analysis to investigate the interactions between cyberbullying experiences and psychological characteristics during this critical period, providing a scientific foundation for developing precise, time-sensitive intervention strategies.

## Methods

### Participants and Data Collection

This study uses data sourced from Sina Weibo, a social media platform developed by Sina Corporation, which had 605 million monthly active users by the end of September 2023. The collected data mainly include users’ web-based behaviors (such as reposts and replies) and Weibo text content. During the data collection process, we adhered to privacy and ethical principles, ensuring that all data were anonymized and privacy was strictly protected [44].

We screened 60 cyberbullying survivors from a pool of Weibo users through a two-step process: (1) preliminary identification of users via public media reports or keyword searches, and (2) manual verification of survivors and determination of the time of their first cyberbullying incident.

#### Step 1: Preliminary Identification of Users

We used 2 approaches to identify users. The first approach involved identifying Weibo users who had been publicly recognized as cyberbullying survivors through media reports. The second approach used keyword searches based on the definition and types of cyberbullying, combined with usage characteristics of the Weibo platform. Keywords included terms such as: “cyber violence” (网络暴力), “cyberbullying” (网暴), “abuse” (辱骂), “defamation” (诽谤), “rumors” (造谣), “insults” (污蔑), “doxxing” (人肉), “mocking” (嘲笑), “harassment” (骚扰), “exclusion” (排斥), “kicked out” (踢出), and “pranks” (恶作剧). Through this process, we initially identified 82 potential Weibo users who might have experienced cyberbullying.

#### Step 2: Manual Verification of Survivors and Determination of Cyberbullying Timing

We recruited 4 graduate students in psychology as research assistants. They were given detailed training on the definitions, types, and manifestations of cyberbullying. The Weibo posts of the 82 initially identified users were downloaded and anonymized. Each research assistant independently reviewed all posts from each user to determine whether they could be classified as a survivor and to identify the time of the user’s first cyberbullying incident. The inclusion criteria were (1) the user had experienced cyberbullying behaviors and (2) the user had expressed in their posts that they perceived those behaviors as harmful. The exclusion criteria were (1) inability to determine the time of the first cyberbullying incident, (2) inability to obtain Weibo posts before and after the first cyberbullying incident, and (3) Weibo users with strong public visibility and influence, such as celebrities. A user was only included as a survivor if at least 3 research assistants agreed on their classification. Ultimately, 60 users were included in the survivor group.

For the control group, we randomly selected active Weibo users (with more than 500 posts) who had not experienced cyberbullying and matched them with the survivors. To ensure effective matching, we used user profiles to match sex, age, location, and number of followers, ensuring no significant differences between the control and survivor groups. The screening and matching process was conducted collaboratively by the same 4 research assistants.

We used the application programming interface provided by Sina Weibo to download the text data of the 120 Weibo users.

### Methods and Measurements

Online ecological recognition (OER) is a technique that uses publicly available web-based social media data combined with machine learning methods to measure corresponding psychological characteristics without contacting participants. Compared to self-reports, OER does not rely on the participants’ self-report results or their cooperation with the experiment, and it does not require any contact with the participants. This makes it a more convenient, safer, and timely method for tracking psychological status [20]. OER mainly involves 3 steps: Chinese word segmentation, lexical feature extraction, and model prediction [45,46]. The predictive models are tools developed based on big data and deep learning techniques for web-based psychological research, and their feasibility has been repeatedly verified by multiple studies [47]. Therefore, this study uses the Oxford Happiness model, SWB model, and PWB model to predict individuals’ happiness; the suicide dictionary to predict individuals’ suicide risk; the personality prediction model to predict individuals’ Big 5 personality traits; the Moral Foundations Dictionary (MFD) and Moral Motivation Dictionary (MMD) to predict moral perceptions; and the Simplified Chinese version of Linguistic Inquiry and Word Count (SC-LIWC) to supplement the prediction of psychological traits.

The models and dictionaries selected for this study are as follows:

- Oxford Happiness model: This study uses this model to predict Oxford Happiness Inventory scores [28], aiming to assess overall levels of individual happiness. Wang et al [48] selected 548 Weibo users as their sample and divided them into high- and low-happiness groups based on their Oxford Happiness Inventory scores. They extracted linguistic and behavioral features from social media data and used a decision tree model to predict participants' happiness levels, achieving an accuracy rate of 0.68.
- SWB model: This model is used to predict emotional well-being levels measured by the Positive and Negative Affect Schedule. Hao et al [49] developed and validated this model using social media data from 1785 Weibo users. The optimal evaluation metrics for the model’s effectiveness in predicting SWB showed a strong correlation level of 0.60.
- PWB model: This model is used to predict scores on the PWB Scale by Ryff and Keyes [27], covering dimensions such as autonomy, environmental mastery, personal growth, positive relations with others, life purpose, and self-acceptance. Han et al [50] developed and validated these machine learning models using the textual features of 1427 Weibo users. The correlation between predicted and self-reported PWB scores was significantly positive across all 6 dimensions.
- Personality prediction model: The study uses this model to estimate scores on the Big 5 personality dimensions: openness, agreeableness, extraversion, conscientiousness, and neuroticism. Han et al [51] developed and validated this machine learning model using textual features from 3886 Weibo users. The model demonstrated good structural and criterion validity, with 5-fold cross-validation results ranging from 0.44 to 0.48 (*P*<.001). The correlation coefficients for the 5 personality traits between the 2 split datasets ranged from 0.84 to 0.88 (*P*<.001).
- Chinese Suicide Dictionary: This study uses this dictionary to predict suicide risk. The dictionary is designed to identify suicide risk on social media, comprising 2168 words divided into 13 categories (eg, suicidal ideation words, hopelessness words, and personality words) [52].
- Moral Dictionary: This dictionary is used to measure changes in the survivors’ morality. It includes MFD [53] and MMD [54]. The simplified Chinese version of the MFD includes 590 words or phrases across 6 dimensions (harm, fairness, loyalty, authority, purity, and general morality), with positive and negative word lists for all dimensions except general morality. The Chinese version of the MMD includes 2 dimensions: agency and communication. The agency includes 690 words (eg, achievement, failure, and expenditure), while communication includes 260 words (eg, acceptance, care, and kindness).
- SC-LIWC: In addition to the earlier models and dictionaries, this study uses SC-LIWC to supplement the analysis of psychological changes in cyberbullying survivors. SC-LIWC is widely used in natural language processing, covering multiple dimensions such as linguistic processes, psychological processes, and personal concerns [55]. This study specifically focuses on the subcategories of “I,” “tPast,” “ tFuture,” “Anger,” “CogMech,” “Insight,” “Cause,” and “Certain.” For example, “I” reflects the degree of self-focus; “Anger” captures the intense emotional responses elicited by bullying; “tPast” and “tFuture” reveal the survivor’s immersion in past experiences and concerns about the future. Changes in these dimensions help elucidate the impact of cyberbullying on survivors’ well-being, suicide risk, and personality traits. Additionally, “CogMech” and “Insight” reflect the survivor’s cognitive processing and self-reflection, while “Cause” and “Certain” indicate the survivor’s perception of causality and uncertainty regarding future events. The analysis of these dimensions further enhances our understanding of the effects of cyberbullying on well-being, suicide risk, personality traits, and moral judgment.

### Data Analyses

The study uses the starting time of the initial cyberbullying incident as the dividing line to measure psychological characteristics before and after this event. The first measurement uses Weibo data from 3 months before the cyberbullying incident, and the second measurement uses data from 3 months after the incident. Given that the timing of the initial cyberbullying incident may vary among users, we estimated each survivor's mental health status precisely three months before and after the event. The control group, consisting of individuals who have not experienced cyberbullying, was analyzed during the same period, with the timeline matched one-to-one.

We conducted descriptive statistics, independent sample 2-tailed *t* tests, and paired sample 2-tailed *t* tests using SPSS (version 27.0; IBM Corp). Initially, we analyzed the between-group differences between the survivor group (n=60) and the control group using independent sample *t* tests. Then, we compared the changes in psychological characteristics at T1 (3 months before the cyberbullying incident) and T2 (3 months after the incident) for both groups using paired sample 2-tailed *t* tests. All analyses were conducted at a significance level of α=.05, and effect sizes of mean differences were reported as *d*.

We used R (version 4.2.2; R Foundation for Statistical Computing) to perform network analysis on the predictive psychological characteristics and cyberbullying experiences at T2, estimating, visualizing, and evaluating the network.

### Ethical Considerations

This study was conducted in accordance with ethical guidelines. The research involved the analysis of anonymized social media data, ensuring that no personally identifiable information was retained.

The study received ethical approval from the institutional review board of Central China Normal University, with the approval number (CCNU-IRB-202311046b), confirming that the research adhered to ethical standards for human subjects research.

Given the nature of the study, which involved the analysis of publicly available, anonymized data, informed consent was not required. The original data collection processes were conducted in compliance with relevant ethical guidelines, and the secondary analysis of this data was covered under the original ethical approval.

All data used in this study were anonymized to protect participant privacy. No personally identifiable information was included in the analysis, and stringent measures were taken to ensure data confidentiality.

No compensation was provided to participants, as the study utilized existing anonymized data and did not involve direct interaction with participants.

## Results

### User Statistics

#### Overview

Among the users who registered their birth date in their profiles (N=120; survivor group n=60, 50%; control group n=60, 50%), 78% (94/120) were female, with ages ranging from 15 to 41 years, and an average age of 28 (SD 6.23) years. The sample included 1 user who died by suicide and another who died of cancer. Table 1 presents the demographic characteristics of these groups.

**Table 1:** Participant demographic characteristics.

			Total (N=120), n (%)		Survivor group (n=60), n (%)		Control group (n=60), n (%)
Number of deaths			2 (2)		2 (3)		0 (0)
**Sex**							
	Male	26 (22)		13 (22)		13 (22)	
	Female	94 (78)		47 (78)		47 (78)	
**Age (years)**							
	Less than 18	6 (5)		3 (5)		3 (5)	
	18-29	84 (70)		42 (70)		42 (70)	
	30-39	22 (18)		11 (18)		11 (18)	
	40-49	8 (7)		4 (7)		4 (7)	

#### The Impact of Cyberbullying on Psychological Characteristics

To investigate the impact of cyberbullying on mental health, we first compared the scores of Oxford Happiness, SWB, PWB, suicide risk, personality traits, and moral perception between the survivor group and the control group at T1. Then, we separately compared the scores of the survivor group and the control group between T1 and T2. At T1, we found no significant differences between the survivor group and the control group in Oxford Happiness, SWB, PWB, suicide risk, personality traits, or moral perception. Specifically, for personality traits, no significant differences were observed in agreeableness (*t*_118_=–0.77; *P*=.45), conscientiousness (*t*_118_=–0.03; *P*=.98), extraversion (*t*_118_=–0.72; *P*=.47), openness (*t*_118_=0.34; *P*=.73), or neuroticism (*t*_118_=1.17; *P*=.25). Additionally, the control group showed no significant changes between T1 and T2. In the survivor group, significant reductions were observed after cyberbullying in Oxford Happiness (*t*_59_=2.14; *P*=.04), positive emotions (*t*_59_=2.72; *P*=.009), positive relations with others (*t*_59_=2.54; *P*=.01), purpose in life (*t*_59_=2.28; *P*=.03), and self-acceptance (*t*_59_=2.08; *P*=.04). Environmental mastery showed a marginally significant decrease (*t*_59_=1.72; *P*=.09), while negative emotions significantly increased (*t*_59_=–2.89; *P*=.005). The “Anger” of SCL-LIWC also significantly increased (**t*_59_*=–3.46; *P*=.001). In the cognitive processes category, the “Certain” of SCL-LIWC significantly increased (*t*_59_=–2.82; *P*=.006), while “Insight” words (*t*_59_=–1.69; *P*=.096) and “Cause” words (*t*_59_=–1.77; *P*=.08) showed marginally significant increases. Regarding the Chinese suicide dictionary, self-regulation showed a marginally significant decrease (*t*_59_=1.75; *P*=.09), while shame and guilt (*t*_59_=–1.71; *P*=.09) and anger and hostility (*t*_59_=–1.80; *P*=.08) marginally increased. Among the Big 5 personality traits, agreeableness (*t*_59_=2.79; *P*=.007), extraversion (*t*_59_=2.26; *P*=.03), and conscientiousness (*t*_59_=2.27; *P*=.03) significantly decreased, while neuroticism significantly increased (*t*_59_=–3.42; *P*=.001). In the MMD and MFD, communication words (**t*_59_*=–2.62; *P*=.011) and FairnessVice (**t*_59_*=–2.20; *P*=.03) significantly increased, while PurityVice showed a marginal increase (**t*_59_*=–1.88; *P*=.07; Table 2).

**Table 2:** The difference in psychological characteristics between the survivor group and the control group at T2 and T1.

Indexes and dimensions				Survivor group (n=60)							Control group (n=60)					
				Mean (SD)		*t* test (*df*)		*P* value		Mean (SD)				*t* test (*df*)		*P* value
**Oxford Happiness**																
	**Oh**					2.14 (59)^a^		.04						0.77 (59)		.45
		Before	87.98 (4.22)						88.01 (4.42)							
		After	86.99 (3.12)						87.67 (3.30)							
**Subjective well-being**																
	**Positive emotion**					2.72 (59)^b^		.009						0.68 (59)		.50
		Before	21.84 (0.91)						21.90 (0.99)							
		After	21.27 (0.68)						21.82 (0.81)							
	**Negative emotion**					–2.90 (59)^b^		.005						–0.71 (59)		.48
		Before	14.54 (1.50)						14.48 (1.43)							
		After	14.98 (1.26)						14.57 (1.04)							
**Psychological well-being**																
	**Environmental mastery**					1.73 (59)^c^		.089						–0.54 (59)		.59
		Before	11.55 (0.68)						11.46 (0.69)							
		After	11.46 (0.66)						11.50 (0.54)							
	**Positive relations with others**					2.54 (59)^a^		.01						0.77 (59)		.45
		Before	12.43 (0.46)						12.54 (0.44)							
		After	12.28 (0.38)						12.50 (0.39)							
	**Autonomy**					0.68 (59)		.50						–0.62 (59)		.54
		Before	11.30 (0.76)						11.24 (0.80)							
		After	11.25 (0.69)						11.29 (0.69)							
	**Personal growth**					1.16 (59)		.25						–0.02 (59)		.99
		Before	15.07 (0.31)						15.01 (0.36)							
		After	15.03 (0.33)						15.01 (0.31)							
	**Self-acceptance**					2.08 (59)^a^		.04						0.37 (59)		.72
		Before	10.10 (0.68)						10.05 (0.72)							
		After	9.98 (0.64)						10.03 (0.59)							
	**Purpose in life**					2.28 (59)^a^		.03						0.18 (59)		.86
		Before	12.85 (0.61)						12.81 (0.61)							
		After	12.72 (0.53)						12.79 (0.51)							
**Personality**																
	**Agreeableness**					2.79 (59)^b^		.007				0.61 (59)				.55
		Before	32.71 (0.84)						32.81 (0.78)							
		After	32.36 (0.98)						32.76 (0.73)							
	**Conscientiousness**					2.27 (59)^a^		.03						0.58 (59)		.56
		Before	28.833 (1.28)						28.84 (1.13)							
		After	28.47 (1.19)						28.76 (1.13)							
	**Extraversion**					2.26 (59)^a^		.03						1.58 (59)		.12
		Before	23.30 (1.09)						23.43 (0.94)							
		After	22.99 (0.93)						23.23 (1.01)							
	**Openness to experience**					1.66 (59)		.10						0.53 (59)		.60
		Before	35.85 (0.97)						35.79 (0.99)							
		After	35.63 (1.09)						35.73 (0.99)							
	**Neuroticism**					–3.42 (59)^d^		.001						–0.97 (59)		.33
		Before	24.53 (1.16)						24.28 (1.23)							
		After	25.03 (1.12)						24.40 (1.31)							
**SC-LIWC^e^**																
	**I**					–2.54 (59)^a^		.01						0.73 (59)		.46
		Before	0.020 (0.10)						0.0138 (0.0098)							
		After	0.023 (0.012)						0.013 (0.0087)							
	**tPast**					–1.77 (59)^c^		.08						–0.62 (59)		.54
		Before	0.0016 (0.0014)						0.0018 (0.0022)							
		After	0.0020 (0.0015)						0.0022 (0.0058)							
	**tFuture**					–2.14 (59)^a^		.04						–1.68 (59)		.10
		Before	0.00078 (0.00074)						0.00086 (0.0010)							
		After	0.0011 (0.0011)						0.0013 (0.0018)							
	**Anger**					–3.46 (59)^d^		.001						0.86 (59)		.39
		Before	0.0019 (0.0014)						0.0029 (0.0035)							
		After	0.0037 (0.0033)						0.0025 (0.0027)							
	**CogMech**					–2.30 (59)^a^		.02						–0.56 (59)		.57
		Before	0.11 (0.032)						0.102 (0.0311)							
		After	0.12 (0.020)						0.104 (0.027)							
	**Insight**					–1.69 (59)^c^		.096						–0.38 (59)		.71
		Before	0.012 (0.0062)						0.011 (0.0056)							
		After	0.014 (0.0054)						0.011 (0.0061)							
	**Cause**					–1.77 (59)^c^		.08						0.89 (59)		.38
		Before	0.0075 (0.0042)						0.0065 (0.0043)							
		After	0.0086 (0.0044)						0.0059 (0.0042)							
	**Certain**					–2.82 (59)^b^		.006						0.72 (59)		.48
		Before	0.010 (0.0055)						0.015 (0.032)							
		After	0.12 (0.0058)						0.011 (0.006)							
**Chinese Suicide Dictionary**																
	**Self-regulation**					1.75 (59)^c^		.09						0.17 (59)		.87
		Before	0.00073 (0.0012)						0.00043 (0.00085)							
		After	0.00045 (0.0007)						0.00041 (0.00084)							
	**Shame and guilt**					–1.71 (59)^c^		.09						0.21 (59)		.84
		Before	0.00050 (0.00066)						0.00071 (0.0011)							
		After	0.00081 (0.0012)						0.00067 (0.0009)							
	**Anger and hostility**					–1.80 (59)^c^		.08						0.17 (59)		.87
		Before	0.0015 (0.0014)						0.0011 (0.0020)							
		After	0.0022 (0.0034)						0.0010 (0.0013)							
**Moral Motivation Dictionary**																
	**C** **ommunication**					–2.62 (59)^a^		.011						1.55 (59)		.13
		Before	0.0052 (0.0032)						0.0046 (0.0041)							
		After	0.0063 (0.0040)						0.0036 (0.0031)							
**Moral Foundations Dictionary**																
	**FairnessVice**					–2.20 (59)^a^		.03						0.82 (59)		.41
		Before	0.000091 (0.00021)						0.000080 (0.00026)							
		After	0.00029 (0.00072)						0.000048 (0.00017)							
	**PurityVice**					–1.87 (59)^c^		.07						–1.27 (59)		.21
		Before	0.00034 (0.00053)						0.00015 (0.00036)							
		After	0.00065 (0.0012)						0.00024 (0.00045)							

^a^There was a significant difference existing with *P*<.05.

^b^There was a significant difference existing with *P*<.01.

^c^There was a significant difference existing with *P*<.1.

^d^There was a significant difference existing with *P*<.001.

^e^SC-LIWC: Simplified Chinese version of Linguistic Inquiry and Word Count.

### Network Analysis Results

#### Network Structure

**Figure 1:**
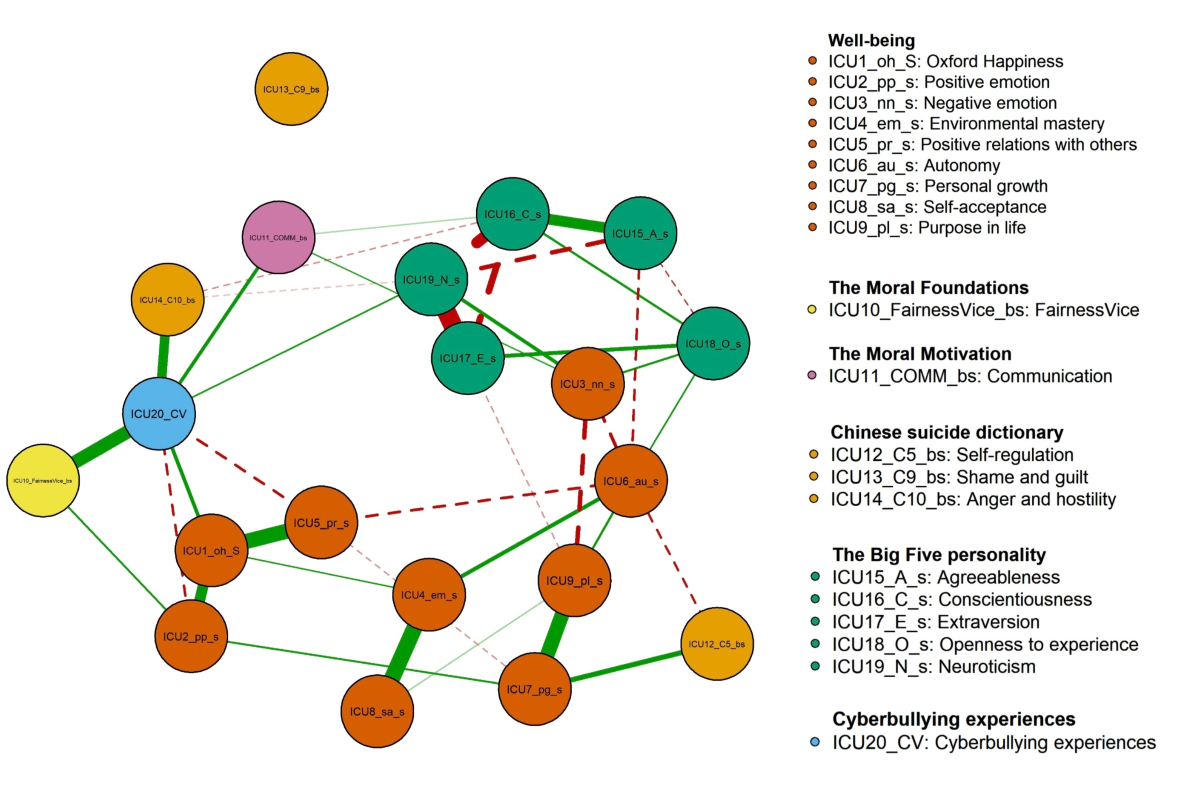
Network structure diagram. ICU1_oh_s: Oxford Happiness; ICU2_pp_s: positive emotion; ICU3_nn_s: negative emotion; lCU4_em_s: environmental mastery; lCU5_pr_s: positive relations with others; ICU6_au_s: autonomy; lCU7_pg_s: personal growth; lCU8_sa_s: self-acceptance; lCU9_pl_s: purpose in life; ICU10_FairnessVice_bs: FairnessVice; ICU11_COMM_bs: communication; ICU12_C5_bs: self-regulation; ICU13_C9_bs: shame and guilt; ICU14_C10_bs: anger and hostility; ICU15_A_s: agreeableness; ICU16_C_s: conscientiousness; ICU17 _E_s: extraversion; ICU18_O_ s: openness to experience; ICU19_N_s: neuroticism; ICU20_CV: cyberbullying experiences.

Figure 1 presents the partial correlation network of the 20 study variables, regularized using the least absolute shrinkage and selection operator. Among the total of 96 edges, 60 were significant, with 35 being positively significant and 25 negatively significant. Network sparsity is 0.68. The edge with the highest weight was between “environmental mastery” and “self-acceptance,” followed by the edge between “personal growth” and “purpose in life,” and the edge between “Oxford Happiness” and “positive relations with others.” The 5 nodes most strongly connected to the “cyberbullying experiences” node were “FairnessVice,” “anger and hostility,” “communication,” “neuroticism,” and “Oxford Happiness,” in descending order of strength.

#### Node Centrality

**Figure 2:**
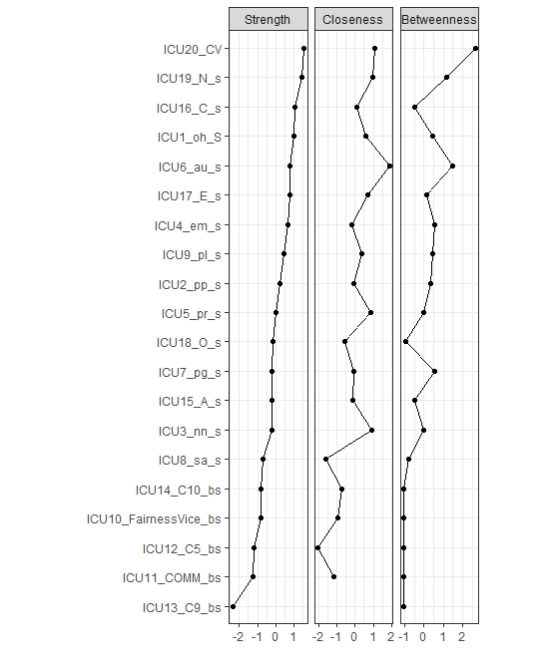
Strength centrality of each node in the network. ICU1_oh_s: Oxford Happiness; ICU2_pp_s: positive emotion; ICU3_nn_s: negative emotion; lCU4_em_s: environmental mastery; lCU5_pr_s: positive relations with others; ICU6_au_s: autonomy; lCU7_pg_s: personal growth; lCU8_sa_s: self-acceptance; lCU9_pl_s: purpose in life; ICU10_FairnessVice_bs: FairnessVice; ICU11_COMM_bs: communication ; ICU12_C5_bs: self-regulation; ICU13_C9_bs: shame and guilt; ICU14_C10_bs: anger and hostility; ICU15_A_s: agreeableness; ICU16_C_s: conscientiousness; ICU17 _E_s: extraversion; ICU18_O_ s: openness to experience; ICU19_N_s: neuroticism; ICU20_CV: cyberbullying experiences.

We report strength centrality, given that betweenness and closeness centrality measures are particularly ill-suited to psychological networks [56]. Figure 2 displays the strength of each node in the network. “Shame and guilt” is the node with the lowest strength, while “cyberbullying experiences” has the highest strength centrality, followed by “neuroticism,” “conscientiousness,” and “Oxford Happiness.” The strength difference test revealed significant differences in strength centrality between “cyberbullying experiences” and “Oxford Happiness,” “autonomy,” “purpose in life,” “conscientiousness,” “extraversion,” and “neuroticism.” The strength centrality of “neuroticism” significantly differed from that of “positive emotions,” “environmental mastery,” “personal growth,” “self-acceptance,” “FairnessVice,” “self-regulation,” “shame and guilt,” “anger and hostility,” “openness to experience,” and “cyberbullying experiences.” Similarly, “Oxford Happiness” exhibited significant strength differences with “positive emotions,” “self-acceptance,” “FairnessVice,” “shame and guilt,” “anger and hostility,“ and “cyberbullying experiences.” The strength of “autonomy” significantly differed from “FairnessVice,” “self-regulation,” “shame and guilt,” “anger and hostility,” “openness to experience,” and “cyberbullying experiences.” Since the strength consistency score (0.517) exceeds the recommended minimum value of 0.25, the reliability of this centrality index is acceptable [57].

## Discussion

### Principal Findings

This study combines the OER method with network analysis to systematically explore the immediate impacts of cyberbullying events on survivors’ internal psychological characteristics and perceptions of the external environment during the pericyberbullying period. The psychological predictive models used in the study, such as Oxford Happiness, have been shown to have good accuracy and precision [23,49-51]. Our research findings indicate that cyberbullying has a significant impact on survivors' psychology within 3 months.

After experiencing cyberbullying, survivors exhibit a significant decrease in internal well-being and positive emotions, while negative emotions significantly increase. They also feel more shame, guilt, anger, and hostility. In terms of personality, there are significant changes in all dimensions of the Big 5 personality traits except for openness. Additionally, survivors perceive more social injustice from the external world, with a notable increase in the FairnessVice in moral foundations and communication words in moral motivation. In contrast, control groups do not show significant changes in well-being, emotions, personality, and morality. Consistent with existing research, after accounting for potential preexisting differences between the control and survivor groups, our results reveal the negative impact of cyberbullying on psychological characteristics [16,40,58-63]. The results of the network analysis indicate that aside from cyberbullying experiences, neuroticism, conscientiousness, and Oxford Happiness exhibit the strongest strength centrality among psychological characteristics, suggesting their critical roles in shaping an individual’s overall psychological state. The high centrality of these nodes implies that altering personality traits and enhancing happiness might be the keys to mitigating the negative impacts of cyberbullying. Previous research has shown that interventions enhancing individual well-being can effectively alleviate the adverse effects of violence experiences [64]. These findings suggest that neuroticism, conscientiousness, and Oxford Happiness serve as core connectors within the psychological network, linking various other psychological variables. Therefore, they play a pivotal role in structuring the network between cyberbullying experiences and psychological characteristics.

Survivors of cyberbullying exhibit marginally significant changes in dimensions related to self-regulation of suicide risk, shame and guilt, and anger and hostility. These emotional and behavioral changes indicate that cyberbullying impacts survivors’ emotional regulation capabilities, increasing their negative emotional experiences [65]. Although these marginally significant changes are not as strong as significant changes, they still suggest that cyberbullying has potentially negative effects on survivors’ mental health and presents a certain suicide risk, warranting further attention and research. The increase in shame and guilt may be related to a decline in self-worth [66], while the increase in anger and hostility may be a reaction to perceived injustice [16].

Regarding the impact of cyberbullying on survivors’ Big 5 personality traits, this study found no significant differences in personality traits between the survivor and the control groups before the occurrence of cyberbullying. This indicates that no specific personality traits predisposed individuals to become survivors of cyberbullying, supporting the absence of a “selection effect” [41] and the notion that anyone can become a survivor of cyberbullying [2]. However, after the occurrence of cyberbullying, survivors exhibited significant changes in their personality traits, with agreeableness, extraversion, and conscientiousness significantly decreasing, while neuroticism significantly increased. These changes reflect the presence of a “socialization effect” [42], indicating that individuals’ personality traits are significantly influenced by the experience of cyberbullying. After experiencing cyberbullying, survivors’ agreeableness and extraversion significantly decreased, suggesting that cyberbullying may cause survivors to withdraw more from social activities and even develop social anxiety disorders [67]. A decrease in conscientiousness could negatively affect their performance at work and school [68], while an increase in neuroticism indicates that survivors become more vulnerable and sensitive in terms of emotional stability [65]. The lack of significant differences in openness before and after cyberbullying may be attributed to the fact that openness primarily reflects an individual's interest in new experiences, art, aesthetics, and abstract thinking, as a more stable trait [69] that is less strongly influenced by short-term environmental factors or external events, such as cyberbullying experiences.

In terms of moral perception of the external environment, the study results show a significant increase in FairnessVice and a marginally significant increase in PurityVice in survivors’ moral foundations, reflecting an increased sensitivity to social injustice. As found by Bai et al [16], cyberbullying experiences may cause individuals’ belief in a just world to collapse. Additionally, there was a significant increase in communicative motivation within survivors’ moral motivations, indicating that survivors may be more inclined to seek support and understanding in interpersonal relationships after experiencing cyberbullying. For example, studies have shown that although survivors may be reluctant to seek help from authorities, they are more likely to seek help from peers [70,71] or web-based support groups [72,73].

The findings of this study have significant practical implications for developing cyberbullying intervention strategies. By focusing on the pericyberbullying period, we can more accurately identify the immediate psychological impacts of cyberbullying on survivors and design more timely and effective intervention measures accordingly. Given the high centrality of nodes such as neuroticism, and Oxford Happiness, these characteristics should be prioritized in interventions aimed at promoting psychological recovery. Although the predictive power of centrality measures has certain limitations for instance, some studies suggest that targeting symptoms based on centrality may be less effective than addressing the most common symptoms [56], strength centrality remains a useful heuristic for identifying key nodes within the network [74]. As core components of the psychological network, improving traits such as Oxford Happiness can generate cascading effects, positively impacting the overall mental health of survivors. This intervention strategy aligns with the principles of positive psychology [25], which emphasizes the importance of happiness, and positive emotions in building psychological resilience and recovering from trauma. Interventions designed to enhance PWB such as acceptance and commitment therapy, have been validated in the recovery of violence survivors, demonstrating their effectiveness in promoting psychological healing [75], and acceptance and commitment therapy have also been shown to effectively reduce neuroticism levels in individuals [76]. By focusing on these key traits, interventions can equip survivors to better manage the emotional challenges posed by cyberbullying and restore a sense of purpose and control in their lives. Regarding changes in the Big 5 personality traits, interventions should focus on increasing agreeableness, extraversion, and conscientiousness in survivors, while mitigating the negative effects of neuroticism. Additionally, to address the increased perception of injustice in the external environment, education and counseling should emphasize helping survivors rebuild their belief in a just world, thereby reducing their negative reactions to cyberbullying. In the future, positive psychology could be incorporated into school curricula to help students develop essential emotional regulation skills for coping with cyberbullying. Schools could also implement group activities or mentorship programs to encourage positive social interactions and establish strong peer and social support systems. Regarding web-based platform management and interventions, platforms could first proactively identify survivors and then offer personalized web-based interventions from a positive psychological perspective to support survivors of cyberbullying.

### Limitations and Future Work

The limitations of this study include a relatively small sample size, with only 60 survivors, although each participant’s Weibo data is substantial. The data source is also limited; to control confounding variables, only survivors from the Weibo platform were studied. Due to the limited personal profile information available on Weibo, we did not consider economic status when matching the experimental group of cyberbullying survivors and the control group of individuals who have not experienced cyberbullying and instead matched participants based on sex, age, location, and number of followers. Additionally, to ensure sufficient Weibo data for accurate predictions of psychological traits, only active users (defined as users with more than 500 posts) were included. Future research could explore the differentiated experiences of cyberbullying across different platforms, varying levels of user activity, and economic status. Additionally, because social media activity decreases for survivors after experiencing cyberbullying [77], we chose a 3-month study period to obtain more data for more accurate predictions of changes in individual psychological characteristics, despite this being a relatively long time frame. Future research could further validate the impact of cyberbullying on survivors’ psychological characteristics by expanding the sample size, incorporating multiple data sources, and examining timely changes in internal psychological characteristics and external perceptions over shorter periods. Moreover, exploring changes in the psychological characteristics of cyberbullying survivors from different cultural backgrounds and age groups, as well as the effects of different types of cyberbullying on psychological characteristics, will also be important directions for future research.

### Conclusions

In conclusion, cyberbullying has a significant negative impact on survivors’ internal psychological characteristics and their perceptions of the external environment. This study leverages social media data and combines OER with network analysis to systematically reveal the dynamic psychological changes in survivors during the pericyberbullying period, before and after the cyberbullying events, as well as the interplay between cyberbullying experiences and psychological characteristics. The findings deepen our understanding of the short-term psychological impacts of cyberbullying and provide a critical theoretical foundation and practical reference for the development of future intervention strategies. Future research and interventions should focus on effectively reducing survivors' neuroticism, enhancing their well-being, and rebuilding their moral cognition to mitigate the negative effects of cyberbullying.

## Abbreviations

MFD: Moral Foundations Dictionary
MMD: Moral Motivation Dictionary
OER: online ecological recognition
PWB: psychological well-being
SC-LIWC: Simplified Chinese version of Linguistic Inquiry and Word Count
SWB: subjective well-being

## Data Availability

Due to privacy concerns, the data used in this study cannot be directly shared in open repositories. However, readers can contact the authors via email to request access to the data.
